# A Multicenter Study on the Relationship of Tumor Lesion Location with Bilateral Parametrial Involvement and Pelvic Lymph Node Metastasis in Cervical Squamous Cell Carcinoma

**DOI:** 10.1245/s10434-024-16802-8

**Published:** 2025-01-25

**Authors:** Xuedong Tang, Baicai Yang, Wei Bian, Kui Li, Shan Pan, Weili Zhu, Weigen Zhou, Anwen Wei, Xiaodong Cheng

**Affiliations:** 1 Department of Gynecologic Oncology, School of Medicine, Women’s Hospital, Zhejiang University, Hangzhou, Zhejiang China; 2 Department of Gynecology, Jiaxing Women and Children’s Hospital Wenzhou Medical University, Jiaxing, Zhejiang China; 3 Department of Radiology, Jiaxing Women and Children’s Hospital Wenzhou Medical University, Jiaxing, China; 4 Department of Radiology, School of Medicine, Women’s Hospital, Zhejiang University, Hangzhou, China; 5 Department of Pathology, Jiaxing Women and Children’s Hospital Wenzhou Medical University, Jiaxing, China

## Abstract

**Background:**

This study aimed to explore the relationship of cervical tumor lesion location (CTLL) with bilateral parametrial involvement (PI) and pelvic lymph node metastasis (LNM).

**Methods:**

The study retrospectively analyzed the clinicopathologic and imaging data of patients with cervical squamous cell carcinoma (SCC) retrieved from multiple centers. According to the CTLL, patients were allocated to three groups: a middle one third group, a unilaterally dominant group, and the entire-region group. Uni- and multivariate logistic regression analyses were performed to explore the preoperative risk factors related to PI and pelvic LNM. The rates of PI and pelvic LNM at the tumor-ipsilateral side and the tumor-contralateral side were compared using the Wilcoxon test.

**Results:**

The study enrolled 776 cases. The CTLL was an important preoperative risk factor for both PI and pelvic LNM. Parametrial involvement occurred solely on the tumor-ipsilateral side (3.57 %) in the unilaterally dominant group, whereas the rate of pelvic LNM on the tumor-ipsilateral side was 11.22 %, significantly higher than on the contralateral side (5.1 %), with no pelvic LNM found on the tumor-contralateral side of patients with tumors smaller than 3.5 cm.

**Conclusions:**

Cervical SCC exhibits the characteristic of more accessible tumor-ipsilateral PI and pelvic LNM. When evaluation by magnetic resonance imaging (MRI) shows that the tumor lesion does not involve the contralateral one third of the cervix, a reduction in the resection scope of the contralateral parametrium can be considered, avoiding resection of the para-aortic lymph nodes, and if the tumor is smaller than 3.5 cm, a reduction in the resection scope of the tumor-contralateral pelvic lymph nodes also can be considered.

Cervical cancer is the fourth most common malignant tumor in women globally. Cervical cancer was diagnosed in approximately 570,000 cases, and 311,000 patients died of the disease in 2018.^1^ Most of these women had cervical cancer diagnosed at an early stage, for which surgery is the standard treatment.^2^

The history of cervical cancer surgery can be traced back to 1895, when Wertheim first proposed extensive hysterectomy, and the operation was later named Wertheim (Piver II). Wertheim advocated removal of the uterus, parametrium, and upper vagina in their entirety and demonstrated that the operation effectively improved the prognosis of cervical cancer patients.

In 1930, Meigs improved Wertheim's operation and expanded the resection scope of the parametrium as he uncovered tumor cells infiltrating into the parametrial lymphatic system in numerous patients. Therefore, Meigs proposed that pelvic lymphadenectomy be performed routinely on the basis of extensive hysterectomy, and his procedure was subsequently termed the Meigs’ operation (Piver III). Because effective cervical cancer screenings at the time were lacking, cervical cancer could not be detected very early, and due to the lack of radiotherapy equipment, the curative effect of adjuvant treatment for cervical cancer could not be ensured. Therefore, the vast majority of scholars advocated for removal of enough parauterine tissue (2 to 3 cm outside the tumor) to ensure a negative margin and reduce recurrence. However, surgically related complications, including urinary system injury, bladder dysfunction, and rectal dysfunction, were subsequently increased.^3,4^ Moreover, serious complications related to lymphatic reflux disorder caused by large-scale systematic pelvic lymphadenectomy were observed (including lower-limb edema and lymph cyst formation), seriously affecting patient quality of life. How to reduce these surgical complications has therefore always been an urgent issue.

Several retrospective studies at the beginning of this century showed a very low risk of parametrial involvement for early-stage cervical patients with favorable pathologic characteristics such as tumor 2 cm in size or smaller, no lymphovascular space invasion (LVSI), and no lymph node metastasis,^5–8^ thus questioning the surgical scope of cervical cancer. Several retrospective studies also showed that less radical surgery for International Federation of Gynecology and Obstetrics (FIGO) 2018 stage IB1 patients did not influence patient overall survival (OS) or disease-free survival (DFS).^9–11^

The results of two prospective studies also depicted no difference in 5-year OS or recurrence rates between patients treated with simple hysterectomy and those undergoing radical hysterectomy.rrence rates between patients treated with simple hysterectomy and those undergoing radical hysterectomy.^12,13^ Investigators conducting the multicenter, prospective, randomized controlled ConCerv and SHAPE trials recently demonstrated the feasibility and oncologic safety of conservative simple hysterectomy surgery for patients with early-stage and low-risk cervical cancer.^14,15^

However, for patients with a larger tumor but with the tumor located on one side of the cervix, we considered whether to reduce the resection scope of the contralateral parametrium and pelvic lymph nodes (PLNs) to reduce the incidence of surgery-related urinary system injury, rectal bladder dysfunction, and lower limb edema.

Previous retrospective studies have focused principally on the risk factors of tumor size, LVSI, deep cervical stromal invasion, and lymphatic metastasis for parametrial involvement (PI) in cervical cancer. We have, however, found no study that examined the relationship between cervical tumor lesion location (CTLL) and bilateral PI or pelvic lymph node metastasis (PLNM). Therefore, in this study we retrospectively analyzed the relationship between CTLL and bilateral PI/PLNM of cervical cancer patients at multicenter hospitals to provide a basis for the individualized and accurate reduction of the scope of surgery.

## Materials and Methods

### Patients and Data Source

This multicenter retrospective study was approved by the Institutional Review Boards and the Ethics Committee of Jiaxing Women and Children’s Hospital of Wenzhou Medical University (approval no. 2024-Y-55). We collected the clinical and pathologic data from Zhejiang University Affiliated Obstetrics and Gynecology Hospital and Jiaxing Women and Children’s Hospital Wenzhou Medical University from March 2021 to March 2024.

The patients were clinically staged using the FIGO 2018 staging system. Our inclusion criteria specified patients with stages IB1 to IIICr cervical cancer who had undergone surgical treatment, had received Q-M type C radical hysterectomy plus pelvic lymphadenectomy with or without para-aortic lymphadenectomy, and had complete postoperative pathologic results and complete preoperative magnetic resonance imaging (MRI) data. Our exclusion criteria ruled out women who had preoperative adjuvant therapy, non-cervical squamous cell carcinoma, cancer in the cervical stump with cervical cancer during pregnancy, cervical cancer found accidentally, manifestation of other malignant tumors, poor MRI imaging, or inconsistent conclusions as to the location of the tumor lesion.

### MRI Localization of Tumor Lesions

Preoperative MRI examinations were performed at 1.5 T (Signa HDxt or Signa Voyager; GE Healthcare, Chicago, IL,USA) using an eight-channel, phased-array cardiac coil. The MRI sequence comprised SAG, AXI, and COR orientations and consisted of the following sequences: fs-T2WI in the sagittal orientation; FLEX-T1WI, T2WI, and DWI in the axial orientation; and dynamic contrast-enhanced MRI with FLEX-T1WI in the axial orientation after injection of Gd-DTPA. Additionally, FLEX-T1WI sequences were performed in the sagittal and coronal orientations.

**Fig. 1:**
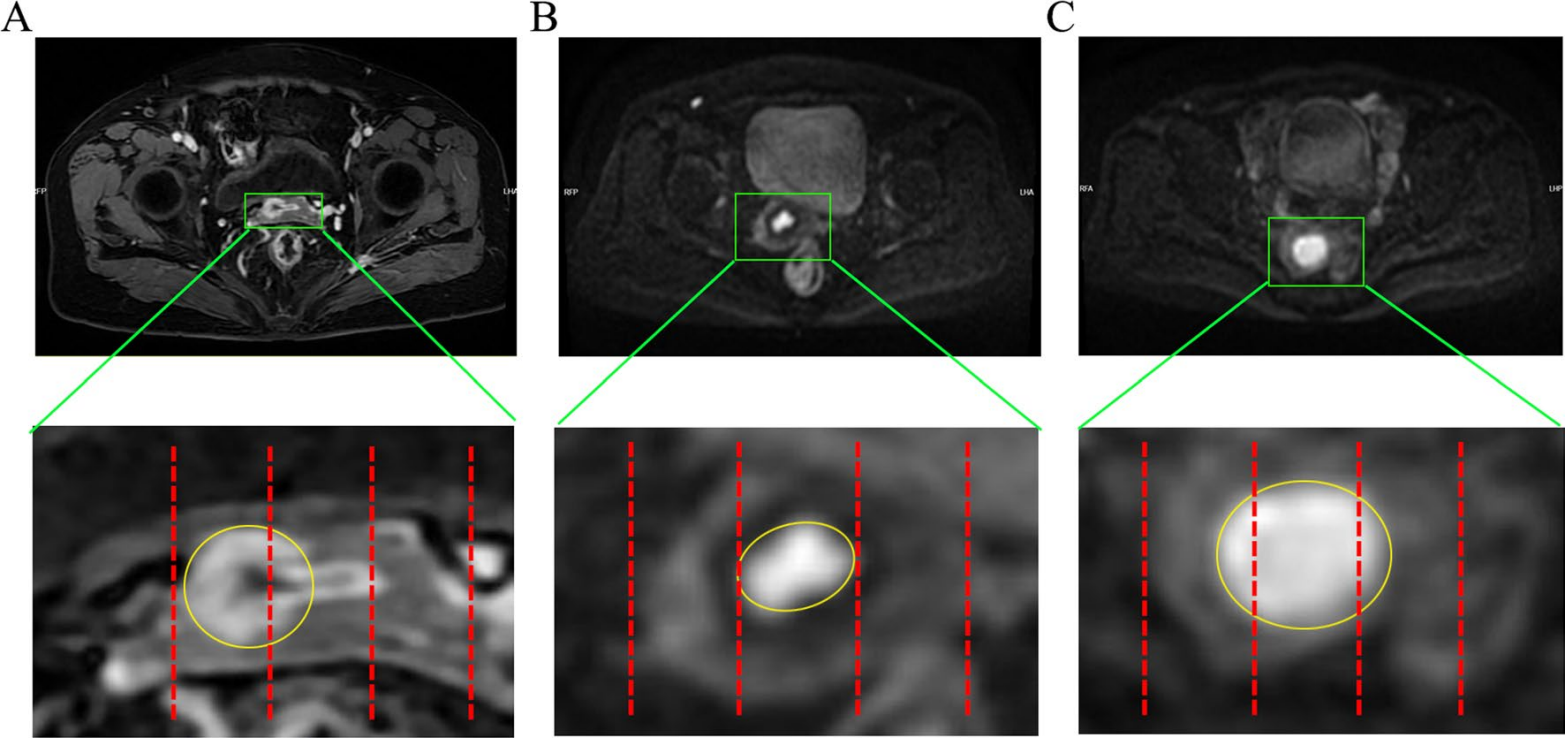
Representative MRI images showing the tumor lesion location. (**A**) Unilaterally dominant group (with the involved tumor in the right third to the middle third of the cervix). (**B**) Middle-third group (with the involved tumor in the middle third of the cervix). (**C**) Entire-region group (with the tumor involving all of the left third, middle third, and right third of the cervix). Red dashed line, longitudinally divided into three equal parts; Oval yellow line, the tumor mass.

The cervix was longitudinally divided into three equal parts on the MRI images: left third, middle third, and right third. According to the CTLL, as shown in Fig 1, the patients were assigned to three groups: a middle third group (with the involved tumor in the middle third of the cervix), a unilaterally dominant group (with the involved tumor in the left third or right third to the middle third of the cervix), and the entire-region group (with the tumor involving all of the left third, middle third, and right third of the cervix).

The cervical cancer lesions had the following MRI features. The tumor displayed intermediate signal intensity on T1-weighted imaging and high signal intensity on T2-weighted imaging. Notably, the tumor exhibited markedly high signal intensity on DWI, whereas the apparent diffusion coefficient (ADC) map showed low signal intensity. Necrosis tended to develop within the tumor as it enlarged. Early enhancement was notably prominent, exceeding the intensity of the surrounding cervical stroma. Conversely, in the late phase, enhancement intensity was lower than that of the normal cervical stroma. The time-signal intensity curve illustrated an outflow pattern.

Preoperative MRI imaging localization of the tumor lesion was independently completed by two radiologists. When the results were inconsistent, a third radiologist joined the evaluation. If the three radiologists were still inconsistent with respect to one another, the case was discarded.

This study compared the differences in clinicopathologic characteristics among the three groups, analyzed the incidence of tumor-ipsilateral and contralateral PI and PLNM of unilaterally dominant lesions, and examined the preoperative risk factors for PI and PLNM.

### Statistical Analysis

We analyzed the data with SPSS 23.0 software (IBM Corp, Armonk, NY, USA). Continuous variables (age and tumor size) are described as mean ± standard deviation, and *t* tests were applied for comparison between the two groups. Categorical variables are described as the frequency (sample rate), and the Pearson chi-square test or Fisher’s exact test was used for comparison of the two groups. We used the Wilcoxon test for comparison of the tumor-ipsilateral and contralateral PI and PLNM. Uni- and multivariate logistic regression analyses were performed to identify the preoperative risk factors. A *P* value lower than 0.05 was regarded as statistically significant for all tests.

## Results

### Clinicopathologic Characteristics of the Three Groups of Patients With Cervical Squamous Cell Carcinoma (SCC)

**Fig. 2:**
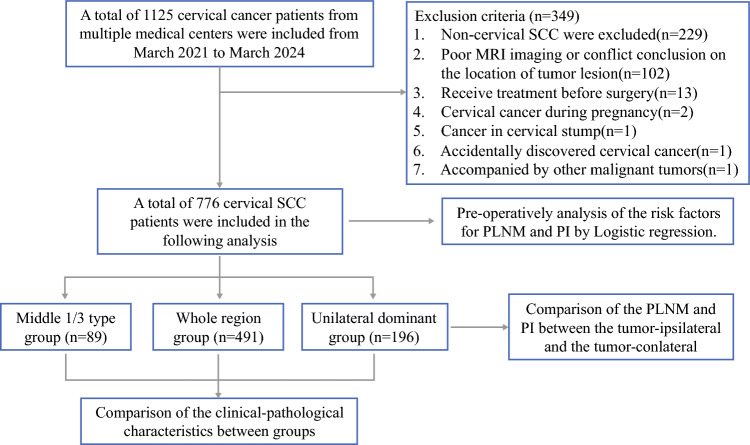
Flowchart of patients included in the analysis. PI, parametrial involvement; CTLL, cervical tumor lesion location; SCC, squamous cell carcinoma. LNM, lymph node metastasis

After strict screening, the study included 776 patients with stage IB1-IIICr: 89 cases in the middle-third group, 196 cases in the unilaterally dominant group, and 491 cases in the entire-region group (Fig 2). As shown in Table 1, the groups did not differ with respect to age or LVSI, but tumor size differed statistically among the three groups. Tumor size was 1.95 ± 0.79 cm in the middle-third group, 2.94 ± 0.98 cm in the unilaterally dominant group, and 4.03 ± 1.17 cm in the entire-region group. We noted no PI in the middle-third group (0/89), and the incidence of PLNM and vaginal involvement (VI) was significantly lower than in the unilateral and entire-region groups. In addition, the rates of VI, PI, and PLNM in both the middle-third group and the unilateral group all were significantly lower than in the entire-region group.

**Table 1:** Clinicopathologic characteristics of cervical SCC patients in three groups

	Middle 1/3 group (*n* = 89) *n* (%)	Unilaterally dominant group (*n* = 196) *n* (%)	Entire-region group (*n* = 491) *n* (%)	P1	P2	P3
Tumor size (cm)	1.95 ± 0.79	2.94 ± 0.98	4.03 ± 1.17	0.000	0.000	0.000
Age(years)	51.88 ± 10.86	52.30 ± 11.94	54.12 ± 11.05	0.771	0.055	0.084
*LVSI*				0.523	0.050	0.026
No	47(52.8)	94(48.0)	195(39.7)			
Yes	42(47.2)	102(52.0)	296(60.3)			
*Nerve involvement*				0.112	0.612	0.029
No	88(98.9)	185(94.4)	456(92.9)			
Yes	1(1.1)	11(5.6)	35(7.1)			
*VI*				0.018	0.018	0.000
No	78(87.6)	147(75.0)	322(65.6)			
Yes	11(12.4)	49(25.0)	169(34.4)			
*PI*				0.103	0.000	0.000
No	89(100.0)	189(96.4)	424(86.4)			
Yes	0(0.0)	7(3.6)	67(13.6)			
*Pelvic LNM*				0.025	0.000	0.000
No	86(96.6)	173(88.3)	374(76.2)			
Yes	3(3.4)	23(11.7)	117(23.8)			
*Para-aortic LNM*				1.000	0.001	0.035
No	89(100.0)	196(100.0)	469(95.5)			
Yes	0(0.0)	0(0.0)	22(4.5)			
*FIGO 2018 stage*						
IB1–IB2	76(85.4)	113(57.7)	155(31.6)	0.000	0.000	0.000
IB3	0(0.0)	19(9.7)	97 (19.8)			
IIA1–IIICr	13(14.6)	64(32.7)	239(48.7)			

*SCC* squamous cell carcinoma, *P1* the middle 1/3 group vs the unilateral dominant group, *P2* the unilateral dominant group vs the whole region group, *P3* the middle 1/3 group vs the whole region group, *LVSI* lymphovascular space invasion. *VI* vagina involvement, *PI* parametrial involvement., *LNM* lymph nodes metastasis, *FIGO* International Federation of Gynecology and Obstetrics

### Analysis of Preoperative Risk Factors for PI and PLNM in Cervical SCC

Logistic regression analysis was implemented to identify the risk factors for PI and PLNM, and our univariate results showed that tumor size, CTLL, FIGO 2018 stage, MRI-determined PI, MRI-determined VI, and MRI-determined PLNM were risk factors for PI. The results of subsequent multivariate analysis showed that only CTLL, FIGO 2018 stage, and MRI-determined PI were independent risk factors for PI (Table 2). Similarly, the risk factors for PLNM were tumor size, CTLL, FIGO 2018 stage, and PI and PLNM as determined radiologically (MRI). Independent risk factors comprised FIGO 2018 stage and MRI-determined PLNM (Table 3).

**Table 2:** Analysis of the preoperative risk factors predicting PI

Risk factors (*n*)	Univariate analysis		Multivariate analysis	
	HR (95 % CI)	*P* Value	HR (95 % CI)	*P* Value
*Tumor size: cm (776)*		0.022		0.882
≤2 (103)	1		1	
2–4 (426)	5.378 (1.279–22.611)	0.022	1.306 (0.290–5.876)	0.728
>4 (247)	7.248 (1.701–30.876)	0.007	1.156 (0.226–5.918)	0.862
*Age: years (776)*		0.318		
≤45 (183)	1			
46–60 (401)	1.612 (0.846–3.072)	0.147		
>60 (192)	1.270 (0.599–2.696)	0.533		
*CTLL (776)*		0.002		0.012
Entire-region group (491)	1		1	
Unilaterally dominant group (196)	0.234 (0.106–0.520)	0.000	0.279 (0.120–0.646)	0.003
Middle 1/3 group (89)	0.000 (0.000–0.000)	0.996	0.000 (0.000–0.000)	0.996
*FIGO 2018 stage (776)*		0.000		0.039
IB1–IB2 (344)	1		1	
IB3 (116)	0.647 (0.214–1.952)	0.439	0.411 (0.113–1.487)	0.175
IIA1–IIICr (316)	3.567 (2.038–6.245)	0.000	1.776 (0.816–3.865)	0.148
*Radiologic PI (776)*		0.000		0.005
No (724)	1		1	
Yes (52)	4.569 (2.370–8.809)	0.000	2.865 (1.385–5.925)	0.005
*Radiologic VI (776)*		0.000		0.808
No (481)	1		1	
Yes (295)	2.477 (1.522–4.031)	0.000	1.079 (0.584–1.994)	0.808
*Radiologic PLNM (776)*		0.000		0.175
No (673)	1		1	
Yes (103)	3.506 (2.034–6.044)	0.000	1.572 (0.818–3.021)	0.175

*PI* parametrial involvement, *HR* hazard ratio, *CI* confidence interval, *CTLL* cervical tumor lesion location, *FIGO* International Federation of Gynecology and Obstetrics, *VI* vagina involvement, *PLNM* pelvic lymph nodes metastasis

**Table 3:** Analysis of the preoperative risk factors predicting PLNM

Risk factors (*n*)	Univariate analysis		Multivariate analysis	
	HR (95 % CI)	*P* Value	HR (95 % CI)	*P* Value
*Tumor size: cm (776)*		0.000		0.866
≤2 (103)	1		1	
2–4 (426)	2.497 (1.046–5.963)	0.039	1.085 (0.407–2.893)	0.871
>4 (247)	7.745 (3.256–18.421)	0.000	1.280 (0.405–4.053)	0.674
*Age: years (776)*		0.134		
≤45 (183)	1			
46–60 (401)	1.034 (0.667–1.602)	0.883		
>60 (192)	0.640 (0.369–1.110)	0.112		
CTLL (776)		0.000		0.108
*Entire-region group (491)*	1		1	
Unilaterally dominant group (196)	0.425 (0.262–0.688)	0.001	0.681 (0.398–1.166)	0.161
Middle 1/3 group (89)	0.112 (0.035–0.359)	0.000	0.295 (0.080–1.095)	0.068
*FIGO 2018 stage (776)*		0.000		0.025
IB1–IB2 (344)	1		1	
IB3 (116)	5.338 (2.940–9.691)	0.000	2.771 (1.217–6.308)	0.015
IIA1–IIICr (316)	5.829 (3.549–9.573)	0.000	2.078 (1.184–3.647)	0.011
*Radiologically PI (776)*		0.000		0.183
No (633)	1		1	
Yes (143)	2.786 (1.534–5.059)	0.001	1.587 (0.804–3.131)	0.183
*Radiologic VI (776)*		0.100		
No (481)	1			
Yes (295)	1.361 (0.942–1.966)	0.100		
*Radiologic PLNM (776)*		0.000		0.000
No (673)	1		1	
Yes (103)	6.866 (4.397–10.722)	0.000	5.148 (3.212–8.253)	0.000

*PLNM* pelvic lymph nodes metastasis, *HR* hazard ratio, *CI* confidence interval, *CTLL* cervical tumor lesion location, *FIGO* International Federation of Gynecology and Obstetrics, *PI* parametrial involvement, *VI* vagina involvement

### Greater Tendency of Unilaterally Dominant Cervical SCC to Show Tumor-Ipsilateral PI and PLNM

According to MRI evaluation, 196 cases of cervical SCC involved patients with unilaterally dominant tumor lesions, among whom seven manifested PI. All of these were in the tumor-ipsilateral parametrium, with none in the tumor-contralateral parametrium (*P* = 0.008; Table 4). Among 26 cases with PLNM, 16 women exhibited tumor-ipsilateral PLNM, 4 women showed tumor-contralateral PLNM, and 6 women manifested both tumor-ipsilateral and tumor-contralateral PLNM (*P* = 0.007; Table 4). When the tumor size was limited to less than 3.5 cm, the incidence of tumor-ipsilateral PLNM was 7 of 98 compared with 0 of 98 tumor-contralateral PLNs (*P* = 0.008; Table 4).

**Table 4:** The PI and PLNM in the unilaterally dominant group

Tumor size (cm)	Any			<3.5		
CTLL	Tumor-ispilateral	Tumor-contralateral	*P* Value	Tumor-ispilateral	Tumor-contralateral	*P* Value
PI	3.57 % (7+0^a^/196)	0 % (0/196)	0.008	5.10 % (5+0^a^/98)	0 % (0/98)	0.025
PLNM	11.22 % (16+6^b^/196)	5.10 % (4+6^b^/196)	0.007	7.14 % (7+0^b^/98)	0 % (0/98)	0.008

*PI* parametrial involvement, *PLNM* pelvic lymph nodes metastasis, *CTLL* cervical tumorlesion location

^a^PI in both tumor-ispilateral and tumor-contralateral

^b^PLNM in both tumor-ispilateral and tumor-contralateral

## Discussion

This study was the first to show a relationship between CTLL and tumor-ipsilateral or tumor-contralateral PI or PLNM. We ascertained that the independent preoperative risk factors for PI in cervical SCC were CTLL, FIGO 2018 stage, and MRI-determined PI, and that the independent risk factors for PLNM were FIGO 2018 stage and MRI-determined PLNM. In addition, we found the characteristic of easier metastasis to tumor-ipsilateral PI and PLNM in cervical SCC. When the unilateral dominance of CTLL was evaluated preoperatively, no PI was observed in the tumor-contralateral parametrium. Thus, clinicians can consider reducing the resection scope of the tumor-contralateral parametrium. When the tumor in these patients was smaller than 3.5 cm, no tumor-contralateral PLNM was detected, and clinicians can therefore also consider reducing the resection scope of the tumor-contralateral PLN.

Several groups have reported that the probability of PI in stage IB1-IIA1 cervical cancer ranged from 4.6 % to 32 %.^16–20^ Kilic et al.^16^ reported that PI was 15.4 % in FIGO stage I and 32.6 % in FIGO stage II. Previous studies also have shown that tumor size, depth of cervical stromal invasion, VI, LVSI(+), and LNM were associated with the PI of stage IB cervical cancer.^21^

These results are mostly consistent with those of the current study. The relationship between histopathologic type and PI, however, remains controversial. Investigators previously discerned that histologic type was associated with PI, but the study by Liang et al.^21^ did not reflect any association in either their uni- or multivariate analysis, and our results were congruent with the latter. However, none of these authors took CTLL into consideration.

We were the first to find that CTLL was an independent risk factor for PI. The incidence of PI in our middle-third group was nil (0/90), significantly lower than the 4.5 % in the unilaterally dominant group (9/200) and the 13.7 % in the entire-region group (69/505). This may have been related to the smaller size and earlier stage of the tumors in the middle-third group. Tumor size in the middle-third group was 1.91 ± 0.68 cm, significantly smaller than in the unilaterally dominant group (2.95 ± 0.98 cm) or the entire-region group (4.03 ± 1.19 cm).

More importantly, we also found that PI occurred in the tumor-ipsilateral parametrium (8/200) rather than in the tumor-contralateral parametrium (0/200), which was in accordance with the biologic characteristics of direct infiltration of cervical SCC. Therefore, our results indicated that we should consider reducing the resection scope of tumor-contralateral parametrium to further reduce the incidence of postoperative complications

Previous studies have shown that the total incidence of PLNM in cervical cancer is 14.8 %, including 4.7 % at stage IA, 13.9 % at stage IB, and 21.0 % at stage IIA,^22^ whereas our study showed that the PLNM rate was 3.6 % (3/83) for the middle-third group, 11.9 % (23/194) for the unilateral group, and 23.0 % (119/598) for the regional group. An earlier report showed that the risk factors for PLNM were tumor size, LVSI, deep cervical stromal invasion, and parametrium and corpus uteri involvement, among which the independent risk factors were tumors larger than 4 cm, LVSI, deep cervical stromal invasion, and corpus uteri involvement.^22–24^ Our results were also principally consistent with these findings.

We additionally demonstrated that preoperatively assessed vaginal involvement was a risk factor for PLNM. However, to our knowledge, no extant study exists on the relationship between CTLL and PLNM. We first reported that CTLL was also a risk factor for LNM, and that it had the characteristics of tumor-ipsilateral PLNM. Our data showed that the probability of tumor-ipsilateral PLNM was 11.3 % (22/194) compared with 5.6 % (11/194) for tumor-contralateral PLNM, with six cases showing bilateral PLNM.

Because tumor size was closely related to PLNM,^23^ we found no PLNM (0/97) in the contralateral pelvic cavity when the tumor volume was further restricted to less than 3.5 cm. This result was consistent with a bilateral lymphatic drainage path for cervical cancer. According to the classical theory, the three following lymphatic drainage paths reach the pelvic lymph nodes in cervical cancer: the anterior path through the bladder cervical ligament, the lateral path through the lateral parametrium, and the posterior path through the uterosacral ligament.^25^ However, the anterior path does not directly receive the drainage of the cervix and participates in the PLNM path of cervical cancer only under specific circumstances such as local lymphatic duct obstruction and retrograde lymph reflux.^26,27^

Although further research is needed to confirm our findings, our data raise the question whether it is possible to narrow the scope of contralateral PLN resection because the probability of lower-limb edema caused by systematic PLN dissection is as high as 47 %.^28^ The severity of lower-limb edema is related to the number and location of the removed PLNs.^28^ If sentinel lymph node biopsy (SLNB) can be used, it may further reduce the occurrence of postoperative complications. The application of SLNB in cervical cancer is supported by high-level medical evidence and has been entered into the diagnosis and treatment guidelines for cervical cancer.

Studies have shown that SLNB is suitable for patients who have early cervical cancer with a primary tumor size of 4 cm or smaller because the rate for detection of SLN is significantly reduced in women with a tumor larger than 4 cm.^29^ Moreover, the rate for detection of SLN in patients with a tumor 2 cm in size or smaller was significantly higher than in individuals with a tumor larger than 2 cm.^30,31^ Therefore, the National Comprehensive Cancer Network (NCCN) guidelines recommend that cervical cancer patients at FIGO 2018 stage IA1 with LVSI, IA2, IB1, and IIA1 may consider undergoing SLNB, and emphasize that although SLNB can be applied to patients with a tumor reaching 4 cm in size, the results of SLNB are more reliable when the tumor is 2 cm in size or smaller.^32^ As for the reason, on the one hand, a larger tumor increases the difficulty of injecting tracer into the cervix. On the other hand, the incidence of PLNM is prominently higher among women with larger cervical tumors, and in cases of metastatic tumor thrombi, tumor compression may cause failure in SLN detection.^33,34^

In the unilaterally dominant group, the aforementioned situation affecting the detection of SLN was nonexistent on the contralateral side of the cervix, and we thus speculated that the SLN detection rate for the tumor-ipsilateral pelvic cavity is likely low, whereas the contralateral side may still have a high detection rate, making it suitable for the application of SLNB. Confirmation of this hypothesis necessitates additional studies.

According to the NCCN guidelines for cervical cancer, MRI is a useful tool for evaluating the stage of cervical cancer preoperatively to optimize clinical treatment due to its significant advantages in soft tissue resolution, tumor size assessment, and evaluation of PI and PLNM. With continuing progress in MRI technology, MRI has shifted from anatomic morphology imaging to functional diffusion-weighted imaging (DWI), which can be used effectively to detect the diffusion movement of water molecules and may produce a diminished tissue signal intensity. The diffusion of water molecules in tumor tissue is limited because of the rapid cellular proliferation, high density, and small extracellular space. The signal intensity in tumor tissue is therefore robust, making DWI more advantageous in evaluating tumor size, relationships with surrounding tissues, and lymph node metastasis. However, when the cervical cancer is in an early stage and the tumor is small, the change in cervical morphology is insignificant, and in this situation, enhanced MRI scanning should be combined with other imaging methods.

A previous study showed that the accuracy of DWI combined with T2-weighted imaging was significantly higher than T2-weighted imaging alone in the preoperative evaluation of cervical cancer.^35^ In the current study, we also applied DWI and T2-weighted imaging to evaluate CTLL, and two or more specialized radiologists independently evaluated location to further improve accuracy.

This study had several advantages. First, we reported the characteristic ease of access with respect to tumor-ipsilateral PI and PLNM in the unilaterally dominant group. We also provided support for narrowing the resection scope of tumor-contralateral parametrium and PLN to reduce postoperative complications.

Second, because this was a multicenter study with a large sample, we expected that the selection bias inherent to a single medical center would be reduced, increasing study credibility. However, this was a retrospective study, and we expect additional prospective studies to confirm our results.
